# Proguanil and atovaquone use is associated with lower colorectal cancer risk: a nationwide cohort study

**DOI:** 10.1186/s12916-022-02643-3

**Published:** 2022-11-10

**Authors:** Naiqi Zhang, Jan Sundquist, Kristina Sundquist, Jianguang Ji

**Affiliations:** 1 Center for Primary Health Care Research, Lund University/Region Skåne, Skåne University Hospital, Jan Waldenströms gata 35, 205 02 Malmö, Sweden; 2 Department of Family Medicine and Community Health, Department of Population Health Science and Policy, Icahn School of Medicine at Mount Sinai, New York, USA; 3 Center for Community-based Healthcare Research and Education (CoHRE), Department of Functional Pathology, School of Medicine, Shimane University, Matsue, Japan

**Keywords:** Proguanil, Atovaquone, Colorectal cancer, Family history, Chemoprevention

## Abstract

**Background:**

Individuals with a family history of colorectal cancer (CRC) are at a high risk of developing CRC. Preclinical studies suggest that the anti-malaria drug proguanil and atovaquone might play a role in preventing CRC, but population-based evidence is still lacking.

**Methods:**

By accessing a couple of nationwide Swedish registers, we performed a cohort study to explore whether using proguanil and atovaquone might associate with a lower risk of CRC by adopting a new-user study design. Adults who have 1 or more first-degree relatives (parents or siblings) diagnosed with CRC were identified and linked with the Prescribed Drug Register to evaluate their administration history of proguanil and atovaquone. Survival analysis of the time to CRC diagnosis with Cox proportional hazards regression was used to estimate hazard ratios (HRs) and 95% confidence intervals (CIs).

**Results:**

A total of 16,817 incident proguanil/atovaquone users were identified and matched with 168,170 comparisons, who did not use proguanil/atovaquone, on the ratio of 1:10. We found a significant negative association between proguanil/atovaquone use and risk of CRC (adjusted HR, 0.76; 95% CI, 0.62–0.93). Test for trend showed significant dose- and duration-response correlations (*P* < 0.001). The association was more pronounced in CRC diagnosed at an advanced stage than at an early stage (adjusted HR, 0.69 vs.0.81).

**Conclusions:**

This national-wide population-based cohort study showed that the use of proguanil and atovaquone was associated with a reduced risk of CRC among individuals with a family history of CRC.

**Supplementary Information:**

The online version contains supplementary material available at 10.1186/s12916-022-02643-3.

## Background

Colorectal cancer (CRC) ranks as the third most frequently occurring cancer and the second leading cause of cancer-related deaths worldwide, causing an estimated 1.8 million new cases and 900,000 deaths worldwide in 2018 [1, 2]. Approximately 70–80% of CRC cases are thought to be attributed to environmental factors, which include cultural, social, and lifestyle practices [3], and the remaining are thought to be due to a heritable component [4, 5]. Numerous studies have established that individuals having first-degree relatives diagnosed with CRC have about 2- to 4-fold lifetime risk of developing CRC compared to those without a family history [6, 7]. Considering the relatively high risk of developing CRC, an effective prevention strategy is highly needed for reducing the incidence of CRC among people with family history.

Drug repositioning, identifying new indications of approved drugs beyond the original medical indication, has become an attractive strategy for cancer treatment and prevention. Biguanides are a class of compounds that exert a wide variety of therapeutic effects. Clinically, biguanides are used to treat diabetes (metformin) and malaria (proguanil) [8]. The anti-proliferative activities of biguanides have aroused extensive attention. Epidemiological and preclinical studies provided evidence of the chemopreventive effect of metformin on several solid tumors [9]. However, the therapeutic concentration for metformin-inducing cell growth arrest is high and arduous which is hard to achieve by conventional administration routes, thus difficult to apply in clinical settings [10]. Proguanil, as a member of the biguanide family, has been reported to have excellent anti-proliferative effects. A study by Lea and co-workers showed that among the biguanides including proguanil, buformin, phenformin, and phenyl biguanide, proguanil has the highest inhibitory ability on bladder and colon cancer cells [11]. Besides, chemical modifications to proguanil were reported to have an even stronger effect on the proliferation and migration of human cancer cell lines [12]. Clinically, proguanil is usually used in combination with another anti-malarial medicine to increase its effectiveness. In Sweden, a fixed-dose combination proguanil/atovaquone is commonly prescribed to achieve better prevention effects against drug-resistant malaria strains. Atovaquone was found to have an anti-tumor activity via inhibition of oxidative phosphorylation in mitochondria, which are a major producer of oxygen radicals [13].

So far, there is no population-based study examining the association between the use of proguanil/atovaquone and CRC. By combing a few nationwide registers in Sweden, we aimed to explore the association between exposure to proguanil and atovaquone and the risk of CRC among people with a family history of CRC. We hypothesized that proguanil and atovaquone play a role in the prevention of CRC.

## Methods

### Data sources

The present nationwide cohort study was approved on February 6, 2013, by the Regional Ethical Review Board in Lund (Dnr 2012/795 and later amendments), Sweden. By linkages to the Swedish Multi-generation Register and the Swedish Cancer Registry, we identified all adults who have 1 or more first-degree relatives (parents or siblings) diagnosed with CRC by using the 10th International Classification of Diseases codes C18, C19, and C20 (*n*=477,582). The Swedish Multi-generation Register consists of data from more than 12 million individuals with information available on their biological parents as well as their siblings of the index persons [14]. It has been used in many previous analyses about the familial risk of various cancer. The Swedish Cancer Registry, which is based on compulsory reports from clinical doctors and pathologists/cytologists, has close to 100% complete coverage of the entire Swedish population.

### Assessment of proguanil/atovaquone use

By linkage to the Swedish Prescribed Drug Register, we further retrieved information on prescriptions of proguanil/atovaquone among people with a family history of CRC according to Anatomical Therapeutic Chemical Classification (ATC) code P01BB51. The Swedish Prescribed Drug Register was created on 1 July 2005 and includes data on all prescribed drugs dispensed at pharmacies covering the entire Swedish population with lower than 0.3% missing data [15]. Each record includes the date of dispensation, ATC code, and defined daily dose (DDD), which is defined as the assumed average maintenance dose per day for a drug for its main indication in adults. We adopted a new-user study design with a washout period of a half year to exclude prevalent proguanil/atovaquone users. The entry date was set as 1 January 2006; thus, individuals prescribed proguanil/atovaquone before January 2006 were excluded (*n*=591).

**Fig. 1:**
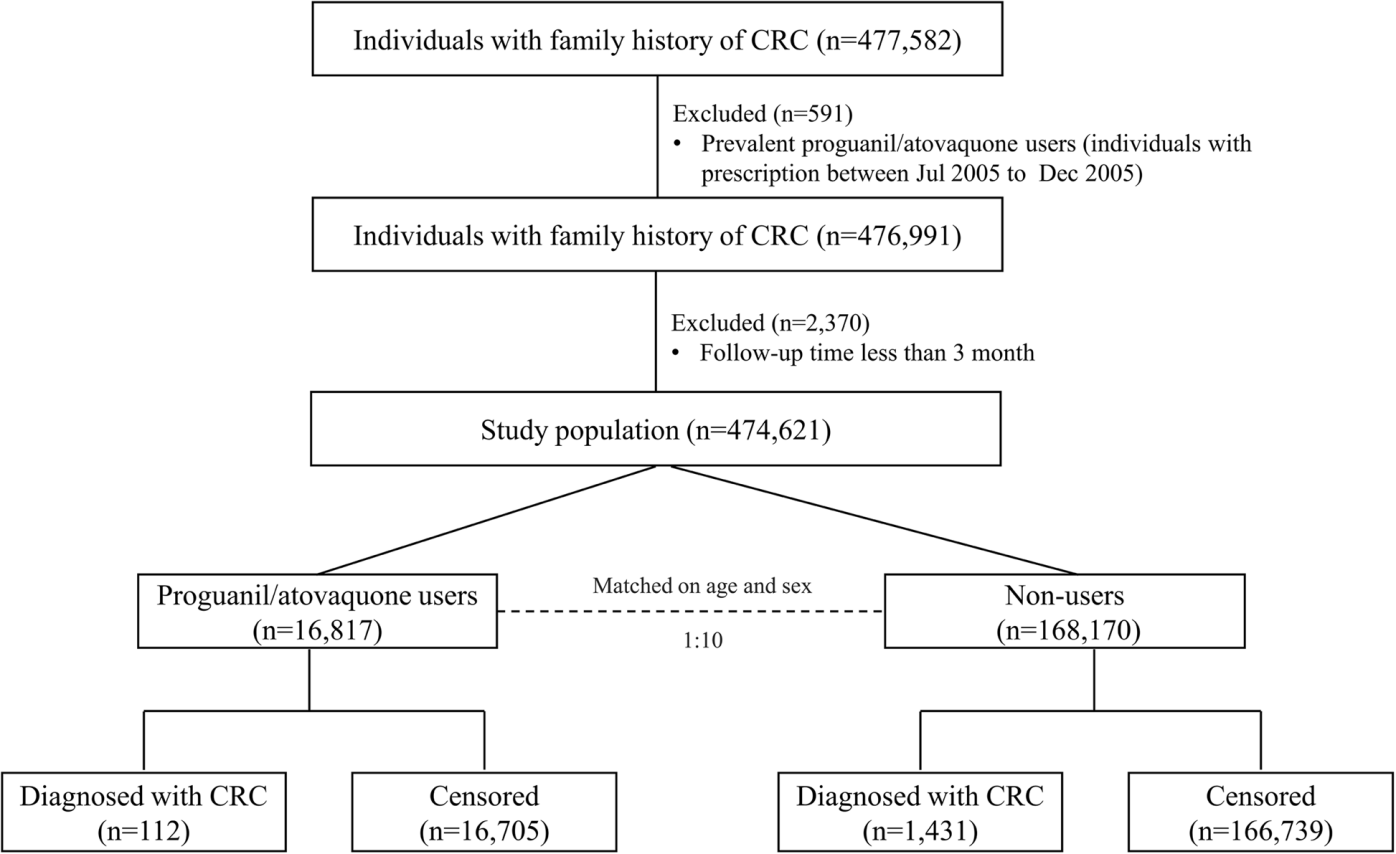
Flow chart of participants involved in this national cohort study

For each proguanil/atovaquone user, up to 10 comparisons who did not receive a prescription of proguanil/atovaquone and had not experienced CRC on the date of the first prescription of the corresponding individual (index date) were randomly selected based on sex and age at index (Fig. 1 for the flowchart of the study design). In Sweden, proguanil/atovaquone was prescribed for three consecutive days when it was used to treat malaria. However, it should be initiated 24 to 48 h before arrival in the malaria-endemic area and continued throughout the stay when it was prescribed as prophylaxis. For those individuals who had multiple prescriptions, the use of proguanil/atovaquone was intermittent. The most common DDD for each prescription was 6 (45.8%) or 3 (33.1%).

### Assessment of outcome

By linkage to the Swedish Cancer Registry, we could identify patients diagnosed with colon or rectal cancer between 1 January 2006 and 31 December 2018 using ICD codes C18, C19, and C20. The Swedish Cancer Registry contains data on the TNM staging system, including the size of the tumor (T), nodal status (N), and presence of metastatic disease (M). By combining the T, N, and M categories, we can determine the stage at diagnosis of CRC, ranging from stage I (the least advanced) to stage IV (the most advanced) as follows: stage I (T1 or T2, N0, M0), stage II (T3 or T4, N0, M0), stage III (any T, N1 or N2, M0), and stage IV (any M1) [16]. By further linkage to the Cause of Death Register, we could identify individuals who had died during the follow-up period, and they were censored during the analyses.

Participants were followed from the date of the first dispensation of proguanil/atovaquone or the index date in the corresponding comparisons and ended at (i) the first date of CRC diagnosis, (ii) the date of death from any cause, and (iii) the end of this study (31 December 2018) whichever came first. To minimize reverse causation, patients with a follow-up time of fewer than 3 months were not included in the study.

### Assessment of covariates

By retrieving data from the National Patient Register and Statistics Sweden’s Total Population Register, we extracted information on potential confounding factors, including age, sex (male or female), birth country (Sweden or others), the highest education level (1–9, 10–11, ≥12 years) [15], history of inflammatory bowel disease (IBD, including Crohn’s disease or ulcerative colitis, yes/no), history of colonoscopy (yes/no), obesity (identified from the National Patient Register using ICD-10 code “E66”, yes/no), chronic obstructive pulmonary disease (COPD, yes/no) as a proxy for smoking, prescription of other medication (statin and statin, yes/no), and Charlson Comorbidity Index score (CCI, 0, 1, 2, ≥3). As comorbidity is an important factor affecting the health condition and risk of cancer, we calculated the CCI based on a total of 17 categories [17]. As there is no national recommendation for the screening of CRC in Sweden, the use of colonoscopy might reflect individuals’ healthy behaviors [18], which might affect our results, we thus included a history of colonoscopy as a confounding factor in our multiple regression models. Individuals with missing values of any variables mentioned above were excluded from the present study.

The Swedish personal identification number was used to link different registers and was then replaced with serial numbers by Statistics Sweden to ensure pseudonymity.

### Statistical analysis

Cox regression models were used to calculate hazard ratios (HRs) and 95% confidence intervals (CIs) of CRC associated with proguanil/atovaquone use. The final multivariable model adjusted for age, sex, birth country, highest education level, history of IBD, colonoscopy, obesity, COPD, use of aspirin, and use of statin. In addition, we calculated the cumulative defined daily doses (cDDDs) of proguanil/atovaquone as the sum of DDDs for all prescriptions during the follow-up period using records from the Swedish Prescribed Drug Register. We then performed a dose-response analysis by modeling cDDD as three tertiles and tested for the trend by entering the median value of the cDDD for each tertile in the regression model. We then evaluated the effect of the duration of proguanil/atovaquone administration. The duration was calculated from the date of the first prescription to the end date of the last prescription. We categorized the proguanil/atovaquone use durations into three groups (<1 week, 1 week to 1 year, ≥1 year). Moreover, we evaluated the association of proguanil/atovaquone use with the risk of the specific site of cancer (colon, rectum) and specific stage of cancer (stage I or II, stage III or IV). We also stratified the analyses based on age and sex to evaluate the interactive effects of proguanil/atovaquone on the risk of CRC.

We further conducted several sensitivity analyses to explore the possibility of chance findings. First, in consideration of biological latency and avoiding reverse causation, we performed a sensitivity analysis lagging the exposure to proguanil/atovaquone for 1 year after the first prescription. Second, we excluded the individuals who had been diagnosed with benign colorectal tumors. Third, we investigated the association of quinine use with CRC risk to evaluate the potential indication bias from proguanil/atovaquone use based on the fact that quinine is also a widely prescribed medication for malaria prophylaxis in Sweden. Fourth, to increase confidence in the reported association, we examined the subsequent risk of accidents, which was used as a negative outcome control. In addition, considering that data were collected concerning a long time interval of 12 years in which the diagnosis and treatment of CRC have changed, we did a stratified analysis by dividing the individuals into two groups according to their index date (group 1: from Jan 1, 2006, to Dec 31, 2011; group 2: from Jan 1, 2012, to Sep 30, 2018), to explore whether there was a difference between the two time period.

All analyses were conducted using SAS, version 9.4 (SAS Institute, Cary, NC).

## Results

Figure 1 shows the flowchart of the study design. A total of 184,987 adults who had at least one first-degree relative diagnosed with CRC were recruited in the present study. Compared with non-users, proguanil/atovaquone users had a higher education level and income, a lower proportion of IBD, obesity, COPD, ever use of aspirin and statin, lower CCI scores, and a higher proportion of colonoscopy history (Table 1). All the variables listed in Table 1 were adjusted for in the final multivariable regression model.

**Table 1:** Demographic and clinical characteristics among proguanil/atovaquone users and matched comparisons

	Proguanil/atovaquone users (*N*=16,817)		Non-users (*N*=168,170)	
	No.	%	No.	%
Age at index				
≤50	6642	39.5	66,420	39.5
51–60	4924	29.3	49,240	29.3
>60	5251	31.2	52,510	31.2
Sex				
Males	8196	48.7	81,960	48.7
Females	8621	51.3	86,210	51.3
Birth country				
Sweden	16,449	97.8	163,114	97.0
Northern European country	122	0.7	2045	1.2
Western European country	50	0.3	489	0.3
Eastern European country	68	0.4	1064	0.6
African country	20	0.1	63	0.1
Middle-Eastern country	50	0.3	784	0.5
Others	58	0.4	611	0.3
Highest education level, *year*				
1–9	1060	6.3	29,582	17.6
10–11	4808	28.6	78,241	46.5
≥12	10,949	65.1	60,347	35.9
Income				
Lowest	2839	16.9	44,167	26.3
Middle-low	3521	20.9	47,135	28.0
Middle-high	3551	21.1	38,168	22.7
Highest	6906	41.1	38,700	23.0
IBD				
No	16,662	99.1	166,063	98.8
Yes	155	0.9	2107	1.2
Obesity				
No	16,681	99.2	165,429	98.4
Yes	136	0.8	2741	1.6
COPD				
No	15,956	94.9	158,059	94.0
Yes	861	5.1	10,111	6.0
Colonoscopy				
No	16,199	96.3	162,277	96.5
Yes	618	3.7	5893	3.5
CCI				
0	14,686	87.3	140,683	83.7
1	1699	10.1	19,707	11.7
2	320	1.9	5053	3.0
≥3	112	0.7	2727	1.6
Outpatient visits, *per year*				
0	9884	58.8	85,803	51.0
1	4959	29.5	48,408	28.8
2	1170	6.9	16,447	9.8
≥3	804	4.8	17,512	10.4
Prescription of other medicines				
Aspirin	1089	6.5	13,243	7.9
Statin	1826	10.9	21,105	12.6

*IBD* inflammatory bowel disease, *COPD* chronic obstructive pulmonary disease, *CCI* Charlson Comorbidity Index score

After an average of 7.1 years of follow-up, the incidence rate of CRC among proguanil/atovaquone users was 9.32 per 10,000 person-years, which was lower than comparisons who did not use proguanil/atovaquone (incidence rate, 12.07 per 10,000 person-years). Proguanil/atovaquone use was inversely associated with CRC risk, with a crude HR of 0.76 (95% CI, 0.63–0.92) and an adjusted HR of 0.76 (95% CI, 0.62–0.93) (Table 2). We observed a dose-dependent effect. The risk of CRC was 0.82 (95% CI, 0.64–1.05) among individuals with the lowest cumulative dose of proguanil/atovaquone (<6 cDDD), decreased to 0.67 (95% CI, 0.44–1.03) among individuals with medium dose (7-12 cDDD), and 0.62 (95% CI, 0.37–1.04) among individuals with the highest cumulative dose (more than 12 cDDD). Similarly, the risk of CRC was decreased with the increase in the duration of proguanil/atovaquone use, with an adjusted HR of 0.84 (95% CI, 0.66–1.09) among individuals with the lowest duration (<1 week), decreased to 0.78 (95% CI, 0.51–1.19) among individuals with medium duration (1 week to 1 year), and 0.57 (95% CI, 0.36–0.90) among individuals with the highest duration (more than 1 year). Tests for the trend of the dose- and duration-response correlation showed significant results (*P* < 0.001).

**Table 2:** Hazard ratios and 95% confidence intervals of colorectal cancer associated with proguanil/atovaquone use among individuals with family history

	Individuals, *n*	Person-years	CRC diagnoses, *n*	IR, per 10,000 person-year	Crude			Adjusted^a^		
					HR	95% CI	*P* value	HR	95% CI	*P* value
Ever use of proguanil/atovaquone										
No	168,170	1,185,525	1431	12.07	1			1		
Yes	16,817	120,203	112	9.32	0.76	0.63–0.92	0.004	0.76	0.62–0.93	0.006
Dose of proguanil/atovaquone										
<6 cDDD	10,748	72,288	72	9.96	0.80	0.63–1.02	0.071	0.82	0.64–1.05	0.112
7–12 cDDD	3729	28,019	24	8.57	0.73	0.48–1.10	0.133	0.67	0.44–1.03	0.070
>12 cDDD	2340	19,896	16	8.04	0.66	0.40–1.10	0.113	0.62	0.37–1.04	0.071
*P* for trend										<0.001
Duration of proguanil/atovaquone										
Less than 1 week	10,642	71,148	72	10.12	0.83	0.66–1.05	0.118	0.84	0.66–1.07	0.155
1week–1year	3188	22,995	21	9.13	0.78	0.52–1.19	0.255	0.78	0.51–1.19	0.248
>1year	2987	26,060	19	7.29	0.63	0.40–0.99	0.046	0.57	0.36–0.90	0.016
*P* for trend										<0.001

*CRC* colorectal cancer, *IR* incidence rate, *HR* hazard ratio, *CI* confidence intervals, *COPD* chronic obstructive pulmonary disease, *IBD* inflammatory bowel disease, *CCI* Charlson Comorbidity Index score

^a^Adjusted for age at index, sex, education, birth country, income, history of inflammatory bowel disease, COPD, obesity, outpatient visits, history of colonoscopy, use of aspirin, use of statin, and CCI

In Table 3, we list the results of different types of CRC associated with proguanil/atovaquone use. We found that the inverse association was statistically significant for colon cancer (adjusted HR, 0.78; 95% CI, 0.61–0.99), but not for rectal cancer (adjusted HR, 0.72; 95% CI, 0.51–1.02). The association was stronger for advanced stages (adjusted HR, 0.69; 95% CI, 0.51–0.92) compared with early stages (adjusted HR, 0.88; 95% CI, 0.65–1.20).

**Table 3:** Subgroup analyses by cancer site and cancer stage

	Individuals, *n*	Person-years	CRC diagnoses, n	IR, per 10,000 person-year	Crude			Adjusted^a^		
					HR	95% CI	*P* value	HR	95% CI	*P* value
Cancer site										
Colon cancer										
Non-users	168,170	1,185,525	933	7.87	1			1		
Users	16,817	120,203	75	6.24	0.78	0.62–0.98	0.003	0.78	0.61–0.99	0.042
Rectal cancer										
Non-users	168,170	1,185,525	498	4.20	1			1		
Users	16,817	120,203	37	3.08	0.72	0.52–1.00	0.049	0.72	0.51–1.02	0.072
Cancer stage										
Stages I and II										
Non-users	168,170	1,185,525	525	4.43	1			1		
Users	16,817	120,203	48	3.99	0.89	0.67–1.17	0.395	0.88	0.65–1.20	0.424
Stages III and IV										
Non-users	168,170	1,185,525	745	6.28	1			1		
Users	16,817	120,203	52	4.33	0.68	0.52–0.89	0.005	0.69	0.51–0.92	0.011

*CRC* colorectal cancer, *IR* incidence rate, *HR* hazard ratio, *CI* confidence intervals, COPD chronic obstructive pulmonary disease, *IBD* inflammatory bowel disease, *CCI* Charlson Comorbidity Index score

^a^Adjusted for age at index, sex, education, birth country, income, history of inflammatory bowel disease, COPD, obesity, outpatient visits, history of colonoscopy, use of aspirin, use of statin, and CCI

In Table 4, we list the results of the stratified analyses. We found that the associations between proguanil/atovaquone use and CRC risk were significant among individuals older than 50 (adjusted HR and 95% CI, 0.75 and 0.61–0.93), but not significant in the age group 18–50 (adjusted HR, 0.81; 95% CI, 0.47–1.40). When stratified by sex, proguanil/atovaquone use was associated with a lower CRC risk among females (adjusted HR, 0.69; 95% CI, 0.59–0.81) compared with males (adjusted HR, 0.83; 95% CI, 0.71–0.98).

**Table 4:** Stratified analyses by age and sex

	Individuals, *n*	Person-years	CRC diagnoses, *n*	IR, per 10,000 person-year	Crude			Adjusted^a^		
					HR	95% CI	*P* value	HR	95% CI	*P* value
Age										
18–50										
Non-users	49,240	508,989	441	8.66	1			1		
Users	4924	51,026	30	5.88	0.83	0.49–1.41	0.497	0.81	0.47–1.40	0.450
>50										
Non-users	101,750	676,536	1251	18.49	1			1		
Users	10,175	69,177	97	14.02	0.75	0.61–0.93	0.007	0.75	0.61–0.93	0.008
Sex										
Male										
Non-users	81,960	580,524	810	13.95	1			1		
Users	8196	59,044	63	10.67	0.75	0.58–0.97	0.031	0.75	0.57–0.97	0.031
Female										
Non-users	86,210	605,001	621	10.26	1			1		
Users	8621	61,159	49	8.01	0.77	0.58–1.03	0.083	0.75	0.56–1.02	0.063

*CRC* colorectal cancer, *IR* incidence rate, *HR* hazard ratio, *CI* confidence intervals, *COPD* chronic obstructive pulmonary disease, *IBD* inflammatory bowel disease, *CCI* Charlson Comorbidity Index score

^a^Adjusted for age at index, sex, education, birth country, income, history of inflammatory bowel disease, COPD, obesity, outpatient visits, history of colonoscopy, use of aspirin, use of statin, and CCI

In Table 5, we list the results of the sensitivity analyses. In sensitivity analysis 1, the use of proguanil/atovaquone continued to be associated with a reduced risk of CRC (adjusted HR, 0.78; 95% CI, 0.63–0.95) when the follow-up was lagged for 1 year after the administration of proguanil/atovaquone. In sensitivity analysis 2, the use of proguanil/atovaquone continued to be associated with a reduced risk of CRC (adjusted HR, 0.77; 95% CI, 0.64–0.93) after excluding individuals with diagnoses of benign colorectal tumors. In sensitivity analysis 3, we found that quinine use was not associated with CRC risk among individuals with a family history of CRC, with an adjusted HR of 0.98 (95% CI, 0.47–2.03). In sensitivity analysis 4, proguanil/atovaquone use was not associated with subsequent accidents (adjusted HR, 1.02; 95% CI, 0.98–1.07). The effect was slightly stronger among individuals recruited from 2012 to 2018 than those recruited from 2006 to 2011 (adjusted HR 0.64 vs. 0.81), but the difference was not statistically significant (*P* for interaction 0.40) (Additional file 1: Supplementary Table).

**Table 5:** Sensitivity analysis

	Individuals, *n*	Person-years	Outcome cases, *n*	IR, per 10,000 person-year	Crude			Adjusted^a^		
					HR	95% CI	*P* value	HR	95% CI	*P* value
Sensitivity analysis 1^b^										
Non-users	161,632	118,0819	1307	11.07	1			1		
Proguanil/atovaquone users	16,202	119,756	104	8.68	0.77	0.64–0.94	0.009	0.78	0.63–0.95	0.016
Sensitivity analysis 2^c^										
Non-users	165,316	1164,939	1394	11.97	1			1		
Proguanil/atovaquone users	16,500	117,889	110	9.33	0.77	0.64–0.93	0.006	0.77	0.64–0.93	0.007
Sensitivity analysis 3^d^										
Non-users	11,350	97,720	119	12.18	1			1		
Quinine users	1135	9615	11	11.44	0.93	0.51–1.66	0.794	0.98	0.47–2.03	0.948
Sensitivity analysis 4^e^										
Non-users	99,040	346,280	22,848	659.81	1			1		
Proguanil/atovaquone users	9904	35,130	2245	639.05	0.97	0.93–1.01	0.084	1.02	0.98–1.07	0.305

*CRC* colorectal cancer, *IR* incidence rate, *HR* hazard ratio, *CI* confidence intervals, *COPD* chronic obstructive pulmonary disease, *IBD* inflammatory bowel disease, *CCI* Charlson Comorbidity Index score

^a^Adjusted for age at index, sex, education, birth country, income, history of inflammatory bowel disease, COPD, obesity, outpatient visits, history of colonoscopy, use of aspirin, use of statin, and CCI

^b^Sensitivity analysis 1: Use of proguanil and the risk of CRC among individuals with a family history of CRC lagging the follow-up time for 1 year after the administration of proguanil/atovaquone

^c^Sensitivity analysis 2: Use of proguanil and the risk of CRC among individuals with a family history of CRC after excluding individuals with benign colorectal tumor

^d^Sensitivity analysis 3: Use of quinine and the risk of CRC among individuals with a family history of CRC

^e^Sensitivity analysis 4: Risk of accidents among those using proguanil/atovaquone. Data on accidents available through December 2012

## Discussion

To our best knowledge, this population-based cohort study based on data from nationwide registers in Sweden is the first study to explore the association between proguanil/atovaquone use and CRC risk. Our study clearly showed that proguanil/atovaquone use was associated with a reduced incidence of CRC among people with a family history of CRC, and the decreased risk of CRC showed dose- and duration-dependent patterns. Besides, we found that the use of proguanil/atovaquone was more strongly associated with CRC diagnosed at an advanced stage (stage III or IV: HR, 0.69) than at an earlier stage (stage I or II: HR, 0.88), suggesting that proguanil/atovaquone might reduce the metastatic potential of cancer cells leading to stage migration.

Individuals with a family history of CRC are at a high risk of developing CRC. Up to 30% of CRC patients have at least one relative with a diagnosis of CRC [19]. Results from systematic reviews consistently show that the risk of developing CRC is 2-fold higher in people with a family history of CRC than in those without a family history and that risk is strongly associated with the number of affected relatives [20, 21] and with a younger age of CRC diagnosis [5]. However, there is limited epidemiological evidence for pharmacological prevention against CRC especially focusing on this high-risk group. The results from the present study suggested that proguanil/atovaquone can protect against the development of CRC, and its chemopreventive effect is even stronger for CRC at an advanced stage.

Mitochondrial metabolism is active and necessary for tumor growth; thus, numerous clinical trials are testing the efficacy of inhibiting mitochondrial metabolism as a new cancer treatment [22]. Although inhibition of complex 1 of the mitochondrial electron transport chain has been the focus of much attention, other sites of action have been considered. Unlike metformin being a complex I inhibitor, proguanil does not inhibit cellular and mitochondrial respiration due to its poor uptake into mitochondria [23]. However, proguanil still displayed the strongest growth inhibition against colon and bladder cancer cells compared to other biguanides including proguanil, buformin, phenformin, and phenyl biguanide. This suggests that proguanil can act on additional extramitochondrial sites inhibiting the proliferation of cancer cells.

Atovaquone has also been observed to have the ability to inhibit mitochondrial respiration in solid tumors. By targeting mitochondrial complex III, atovaquone can reduce oxygen consumption rate and alleviate hypoxia, thus subsequently downregulate mitochondrial respiration, leading to potent inhibition of growth, survival, and migration in FaDu (hypopharyngeal carcinoma), HCT116 (colorectal carcinoma), and H1299 (lung carcinoma) cell lines [24]. Since the hypoxic tumor microenvironment is a common feature of solid tumors, the effect of decreasing the oxygen consumption rate could subsequently render the tumors more sensitive to radiotherapy, thus improving clinical outcomes [25]. A similar growth inhibition effect was also found in other cancers, such as breast [13], cervical [26], thyroid [27], and kidney cancers [28], and retinoblastoma [24]. Based on the preclinical evidence discussed above as well as the evidence from our population-based study, the chemopreventive effect of proguanil and/or atovaquone on CRC is reasonable and feasible, which calls for further randomized clinical trials to confirm their clinical use.

So far, the most promising chemopreventive agent against CRC was aspirin. Low-dose aspirin use was recommended by the United States Preventive Services Task Force (USPSTF) for the primary prevention of CRC in individuals aged 50 to 59 in 2007 [29]. However, the net benefit of aspirin was generally smaller in the updated analysis in 2022 [30]. Besides, the use of aspirin might increase the risk of gastrointestinal bleeding and intracranial hemorrhage, which also underlines the remaining uncertainty about aspirin’s effects when used for primary prevention. So we aim to find a potential chemopreventive agent that both have a good efficacy and safety profile. In general, proguanil/atovaquone is well tolerated and has an excellent safety profile with very rare adverse events during both prophylaxis and treatment courses [31]. A few adverse side effects were reported, including anorexia, nausea, vomiting, abdominal pain, diarrhea, headache, dizziness, and coughing, and most of them were minor and short-affected [32]. Only two serious cases (vanishing bile duct syndrome and anaphylaxis) were reported [33, 34]. Results from our study suggest that the protective effect of proguanil/atovaquone against CRC showed dose- and duration-dependent patterns and significantly reduced the CRC risk in individuals with a dose of more than 12cDDD and duration of more than 1 year. Future well-designed randomized clinical trials are highly needed to establish the effective dosage as well as the safety profile before proguanil/atovaquone can be used as a chemopreventive agent.

The major strength of the current study was that it was done based on the population at a national level, which could efficiently minimize the selection bias and ensure enough statistical power. The cohort study design avoided recall bias and reverse causality. Register-based data also provided us with information on potential demographic and clinical confounding factors. The design also allowed the assessment of dose-response effects of proguanil/atovaquone exposure on CRC. A few limitations warrant consideration. First, the results might be affected by detection bias because individuals who take anti-malaria drugs before traveling to the epidemic area may care more about their health conditions; thus, they may be more likely to screen for CRC and might be diagnosed with CRC at an earlier stage. However, we have adjusted for colonoscopy in our regression model, which is offered in Sweden in an opportunistic manner and can be seen as an indication of high levels of health literacy since people with higher levels of knowledge and higher health awareness are more likely to have colonoscopy [18]. Therefore, the adjustment of colonoscopies might partly exclude the contribution of different health behaviors among individuals who use proguanil/atovaquone and those who do not. Our stratification analyses showed that the use of proguanil/atovaquone was more strongly associated with CRC diagnosed at an advanced stage than at an earlier stage, suggesting such detection bias might play a small role in our observations. Second, we lack information on some potential confounding factors, such as smoking, alcohol drinking, and dietary factors in our nationwide databases. However, we have adjusted for COPD as a proxy of smoking in regression models. Although it is a crude proxy for smoking, it might partly exclude the confounding effect of smoking. We additionally adjusted for education status and income, which are highly associated with lifestyle factors and might partly account for their confounding effect.

## Conclusions

In summary, this population-based cohort study suggests that the use of proguanil/atovaquone is associated with a decreased risk of CRC among people with a family history of CRC, and the decrease showed a dose-dependent relationship. Findings from this study need to be confirmed by well-designed randomization clinical studies in the future.

## Supplementary Information

**Additional file 1: Supplementary table**. Subgroup analysis by index date.

## Data Availability

The data based on the Swedish register are not publicly available due to Swedish law and protecting patients’ privacy, and the combined set of data used for the analysis presented in this study can only be made available from the appropriate Swedish authorities (the Swedish National Board of Health and Welfare (https://www.socialstyrelsen.se/en) and Statistics Sweden (https://www.scb.se/en)), for researchers who meet the criteria for access to confidential data.

## Abbreviations

ATC: Anatomical Therapeutic Chemical Classification
CCI: Charlson Comorbidity Index score
cDDD: Cumulative defined daily dose
CI: Confidence intervals
COPD: Chronic obstructive pulmonary disease
CRC: Colorectal cancer
HR: Hazard ratio
IBD: Inflammatory bowel disease
IR: Incidence rate
USPSTF: The United States Preventive Services Task Force

## References

[1] Bray F, Ferlay J, Soerjomataram I, Siegel RL, Torre LA, Jemal A (2018). Global cancer statistics 2018: GLOBOCAN estimates of incidence and mortality worldwide for 36 cancers in 185 countries. CA Cancer J Clin.

[2] Global Burden of Disease Cancer C, Fitzmaurice C, Abate D, Abbasi N, Abbastabar H, Abd-Allah F, et al. Global, regional, and national cancer incidence, mortality, years of life lost, years lived with disability, and disability-adjusted life-years for 29 cancer groups, 1990 to 2017: a systematic analysis for the global burden of disease study. JAMA Oncol. 2019;5(12):1749–68. 10.1001/jamaoncol.2019.2996 PMC6777271 31560378

[3] Boyle P, Langman JS (2000). ABC of colorectal cancer: epidemiology. BMJ..

[4] Lichtenstein P, Holm NV, Verkasalo PK, Iliadou A, Kaprio J, Koskenvuo M (2000). Environmental and heritable factors in the causation of cancer--analyses of cohorts of twins from Sweden, Denmark, and Finland. N Engl J Med.

[5] Johns LE, Houlston RS (2001). A systematic review and meta-analysis of familial colorectal cancer risk. Am J Gastroenterol.

[6] Johnson CM, Wei C, Ensor JE, Smolenski DJ, Amos CI, Levin B (2013). Meta-analyses of colorectal cancer risk factors. Cancer Causes Control.

[7] Bretthauer M (2011). Colorectal cancer screening. J Intern Med.

[8] Kathuria D, Raul AD, Wanjari P, Bharatam PV (2021). Biguanides: species with versatile therapeutic applications. Eur J Med Chem.

[9] Lv Z, Guo Y (2020). Metformin and its benefits for various diseases. Front Endocrinol (Lausanne).

[10] Barbieri F, Verduci I, Carlini V, Zona G, Pagano A, Mazzanti M (2019). Repurposed biguanide drugs in glioblastoma exert antiproliferative effects via the inhibition of intracellular chloride channel 1 activity. Front Oncol.

[11] Lea MA, Kim H, des BC. (2018). Effects of biguanides on growth and glycolysis of bladder and colon cancer cells. Anticancer Res.

[12] Xiao D, Lu Z, Wang Z, Zhou S, Cao M, Deng J (2020). Synthesis, biological evaluation and anti-proliferative mechanism of fluorine-containing proguanil derivatives. Bioorg Med Chem.

[13] Fiorillo M, Lamb R, Tanowitz HB, Mutti L, Krstic-Demonacos M, Cappello AR (2016). Repurposing atovaquone: targeting mitochondrial complex III and OXPHOS to eradicate cancer stem cells. Oncotarget..

[14] Ekbom A (2011). The Swedish multi-generation register. Methods Mol Biol.

[15] Ji J, Sundquist J, Sundquist K (2018). Cholera vaccine use is associated with a reduced risk of death in patients with colorectal cancer: a population-based study. Gastroenterology..

[16] Brenner H, Kloor M, Pox CP (2014). Colorectal cancer. Lancet..

[17] Quan H, Li B, Couris CM, Fushimi K, Graham P, Hider P (2011). Updating and validating the Charlson comorbidity index and score for risk adjustment in hospital discharge abstracts using data from 6 countries. Am J Epidemiol.

[18] Chen CC, Basch CE, Yamada T (2010). An evaluation of colonoscopy use: implications for health education. J Cancer Educ.

[19] Kerber RA, Neklason DW, Samowitz WS, Burt RW (2005). Frequency of familial colon cancer and hereditary nonpolyposis colorectal cancer (Lynch syndrome) in a large population database. Familial Cancer.

[20] Taylor DP, Burt RW, Williams MS, Haug PJ, Cannon-Albright LA (2010). Population-based family history-specific risks for colorectal cancer: a constellation approach. Gastroenterology..

[21] Butterworth AS, Higgins JP, Pharoah P (2006). Relative and absolute risk of colorectal cancer for individuals with a family history: a meta-analysis. Eur J Cancer.

[22] Vasan K, Werner M, Chandel NS (2020). Mitochondrial metabolism as a target for cancer therapy. Cell Metab.

[23] Bridges HR, Jones AJ, Pollak MN, Hirst J (2014). Effects of metformin and other biguanides on oxidative phosphorylation in mitochondria. Biochem J.

[24] Ashton TM, Fokas E, Kunz-Schughart LA, Folkes LK, Anbalagan S, Huether M (2016). The anti-malarial atovaquone increases radiosensitivity by alleviating tumour hypoxia. Nat Commun.

[25] Tharmalingham H, Hoskin P (2019). Clinical trials targeting hypoxia. Br J Radiol.

[26] Tian S, Chen H, Tan W (2018). Targeting mitochondrial respiration as a therapeutic strategy for cervical cancer. Biochem Biophys Res Commun.

[27] Lv Z, Yan X, Lu L, Su C, He Y (2018). Atovaquone enhances doxorubicin’s efficacy via inhibiting mitochondrial respiration and STAT3 in aggressive thyroid cancer. J Bioenerg Biomembr.

[28] Chen D, Sun X, Zhang X, Cao J (2018). Targeting mitochondria by anthelmintic drug atovaquone sensitizes renal cell carcinoma to chemotherapy and immunotherapy. J Biochem Mol Toxicol.

[29] Force USPST (2007). Routine aspirin or nonsteroidal anti-inflammatory drugs for the primary prevention of colorectal cancer: U.S. Preventive Services Task Force recommendation statement. Ann Intern Med.

[30] Dehmer SP, O'Keefe LR, Grossman ES, Maciosek MV. Aspirin use to prevent cardiovascular disease and colorectal cancer: an updated decision analysis for the U.S. Preventive Services Task Force. Rockville (MD): Agency for Healthcare Research and Quality (US). 2022. Report No.: 21-05283-EF-2. 35648884

[31] Boggild AK, Parise ME, Lewis LS, Kain KC (2007). Atovaquone-proguanil: report from the CDC expert meeting on malaria chemoprophylaxis (II). Am J Trop Med Hyg.

[32] Taylor WR, White NJ (2004). Antimalarial drug toxicity: a review. Drug Saf.

[33] Abugroun A, Colina Garcia I, Ahmed F, Potts S, Flicker M. The first report of atovaquone/proguanil-induced vanishing bile duct syndrome: case report and mini-review. Travel Med Infect Dis. 2019;32:101439. 10.1016/j.tmaid.2019.06.010 31238106

[34] Looareesuwan S, Chulay JD, Canfield CJ, Hutchinson DB (1999). Malarone (atovaquone and proguanil hydrochloride): a review of its clinical development for treatment of malaria. Malarone Clinical Trials Study Group. Am J Trop Med Hyg.

