# Supplementary material for: Proguanil and atovaquone use is associated with lower colorectal cancer risk: a nationwide cohort study
Source: BMC Med. 2022 Nov 10;20:439. doi: 10.1186/s12916-022-02643-3 (PMC9650910; doi:10.1186/s12916-022-02643-3)
Supplement: Supplementary file 1 — Additional file 1: Supplementary table. Subgroup analysis by index date. [file 12916_2022_2643_MOESM1_ESM.docx]

**Supplementary table.** Subgroup analysis by index date.

|  | Individuals, n | Person-years | Outcome cases, n | IR, per 10000 person-year | Crude | | | Adjusted* | | |
| --- | --- | --- | --- | --- | --- | --- | --- | --- | --- | --- |
|  |  |  |  |  | HR | 95% CI | *P* value | HR | 95% CI | *P* value |
| Index date |  |  |  |  |  |  |  |  |  |  |
| 2006-2011 |  |  |  |  |  |  |  |  |  |  |
| Non -users | 91840 | 897024 | 1061 | 11.83 | 1 |  |  | 1 |  |  |
| Proguanil/atovaquone users | 9184 | 91090 | 88 | 9.66 | 0.81 | 0.65-1.00 | 0.051 | 0.81 | 0.65-1.01 | 0.062 |
| 2012-2018 |  |  |  |  |  |  |  |  |  |  |
| Non-users | 76330 | 288500 | 370 | 12.82 | 1 |  |  | 1 |  |  |
| Proguanil/atovaquone users | 7633 | 29113 | 24 | 8.24 | 0.64 | 0.42-0.96 | 0.033 | 0.64 | 0.42-0.97 | 0.037 |
| *P* for interaction |  |  |  |  |  |  |  |  |  | 0.398 |

CRC, colorectal cancer; IR, incidence rate; HR, hazard ratio; CI, confidence intervals; COPD, chronic obstructive pulmonary disease; IBD, inflammatory bowel disease; CCI, Charlson Comorbidity Index score.

*Adjusted for age at index, sex, education, birth country, income, history of inflammatory bowel disease, COPD, obesity, outpatient visits, history of colonoscopy, use of aspirin, use of statin, CCI.
